# Seeing the big picture: imaging in immunotherapy

**DOI:** 10.1093/immadv/ltag024

**Published:** 2026-10-16

**Authors:** Doreen Lau

**Affiliations:** Centre for Inflammation Research and Translational Medicine, Department of Biosciences, Brunel University of London, London, United Kingdom; Department of Radiology, University of Cambridge, Cambridge, United Kingdom

**Keywords:** molecular imaging, theranostics, immunotherapy, positron emission tomography (PET), magnetic resonance imaging (MRI)

Immunotherapies have transformed the treatment of cancer and are increasingly reshaping the management of autoimmune and infectious diseases. By modulating the immune system, these therapies have opened new opportunities to restore protective immunity and overcome immune dysfunction. Despite remarkable clinical successes, therapeutic responses remain highly variable, with many patients failing to respond or developing acquired resistance over time [1]. Understanding the dynamic interplay between immune cells, diseased tissues, and therapeutic interventions has therefore become one of the defining challenges in immunology. As the field continues to evolve, there is an increasing need for technologies that not only characterize immune phenotypes but also enable us to see the ‘big picture’ of *when*, *where*, and *how* immune responses develop, evolve, and determine therapeutic outcome.

For decades, our understanding of immunobiology has relied on molecular, histological, and cellular analyses that have provided invaluable mechanistic insights into immune function. However, these approaches are inherently constrained by endpoint measurements that capture only static snapshots of highly dynamic biological processes. As immunology increasingly embraces spatial biology and systems-level approaches, there is growing recognition that understanding immunity requires moving beyond cataloguing individual cell states and molecular pathways towards considering the immune system as an integrated physiological network operating across tissues and organs [2]. Immune responses are shaped not only by molecular and cellular composition but also by their spatial organization, temporal evolution, and interactions across the whole living organism. Understanding these relationships, immune cell trafficking, and the biodistribution, target engagement, and pharmacodynamics of immunotherapeutics requires technologies capable of monitoring biological processes repeatedly over time and across multiple biological scales.

While microscopy and intravital imaging have revolutionized our understanding of immune cell behaviour and cell–cell interactions within their native tissue microenvironment, they are inherently limited by fields of view, imaging depth, duration of observation, and tissue accessibility. Consequently, these methods cannot readily capture systemic immune responses, longitudinal whole-body biodistribution, or the complex interactions between multiple organs that increasingly underpin immunotherapy efficacy and immune-related toxicity. Bridging these spatial and temporal scales therefore requires molecular imaging approaches that enable immune processes to be studied dynamically, quantitatively, and non-invasively throughout disease and treatment.

## Illuminating the unanswered questions in immunotherapy

Molecular imaging enables the non-invasive visualization, characterization, and measurement of biological processes at the molecular and cellular level in living systems [3]. By combining advances in molecular biology, imaging physics, chemistry, bioengineering, and computational analysis, it provides a complementary approach for interrogating immune responses dynamically across space and time. Unlike conventional endpoint analyses, molecular imaging enables biological processes to be followed longitudinally within the same individual, generating mechanistic insights while facilitating the development of clinically translatable imaging biomarkers. In doing so, molecular imaging is expanding the immunologist’s toolbox and opening new opportunities to address longstanding questions in immunology and immunotherapy.

Molecular imaging is enabling researchers and clinicians to investigate questions that were previously difficult, or impossible, to address using conventional approaches:

- Where do immune cells migrate following immunotherapy, and how do they traffic between organs and tissues?
- When are immune responses initiated, sustained, or exhausted during disease progression and treatment?
- How do immune cells interact with diseased cells and other components of tissue microenvironments?
- Which cellular and molecular processes govern immune activation, regulation, and therapeutic response?
- How do tissue architecture, spatial organization, and cellular heterogeneity influence therapeutic efficacy?
- Can imaging distinguish immune activation from disease progression or treatment-related inflammation?
- How can the pharmacokinetics, biodistribution, target engagement, and pharmacodynamics of immunotherapeutics be monitored non-invasively?
- Can imaging biomarkers predict treatment response, resistance, or immune-related adverse events and guide patient stratification and treatment selection?
- How can imaging accelerate drug development and bridge preclinical studies with clinical trials?

These questions highlight the growing role of molecular imaging in immunology, from visualization towards mechanistic discovery, biomarker development, and precision medicine. The contributions in this Special Collection illustrate how these advances are beginning to transform immunotherapy across the continuum from basic research to clinical practice.

## The special collection: molecular imaging in immunotherapy and image-guided drug delivery

To showcase how molecular imaging is beginning to address these emerging challenges, we assembled this Special Collection on *Molecular Imaging in Immunotherapy and Image-guided Drug Delivery*. Bringing together experts working at the interface between immunology, imaging science, chemistry, engineering, and clinical medicine, the collection showcases technologies that are offering new insights into immunobiology and broadening the applications of molecular imaging in immunotherapy. The contributions illustrate the breadth of today’s molecular imaging toolbox, spanning mechanistic interrogation of immunobiology, clinically translatable imaging biomarkers, image-guided drug delivery, and theranostic strategies.

A central theme is the importance of imaging in deepening our mechanistic understanding of immune responses. Antigen processing and presentation underpin adaptive immunity and determine the success of many immunotherapies by orchestrating T-cell recognition and activation. Lau and Elliott [4] highlight how multiscale and multimodality imaging approaches are providing new insights into these complex pathways, from intracellular antigen processing and peptide presentation to immune synapse formation and T-cell interactions. The review also considers how imaging biomarkers targeting these pathways may facilitate patient stratification and treatment monitoring.

Building upon these mechanistic foundations, advances in imaging physics, radiochemistry, bioengineering, and probe development are rapidly increasing the repertoire of immune-targeted molecular imaging agents. Devoogdt and colleagues [5] provide an overview of radiolabelled nanobody tracers, describing their unique pharmacokinetic properties, high target-to-background ratios, and potential for imaging tumour antigens, immune checkpoints, and immune cell populations in preclinical and clinical settings. As the portfolio of immune-targeted tracers expands, selecting the appropriate imaging strategy for a given biological or clinical question becomes equally important. Heskamp and colleagues [6] address this through a head-to-head comparison of PET imaging approaches for assessing tumour CD8^+^ T-cell infiltration. These studies emphasize that technological innovation must be accompanied by careful biological validation and comparative evaluation to maximize the clinical utility of molecular imaging tools.

The clinical translation of molecular imaging is exemplified by Aarntzen and colleagues [7], who demonstrate the feasibility of immuno-PET using zirconium-89-labelled avelumab in patients with resectable non-small cell lung cancer. Their study shows how molecular imaging can characterize the tumour immune microenvironment non-invasively and identify imaging biomarkers associated with therapeutic response. More broadly, radiolabelled therapeutic antibodies provide valuable opportunities to evaluate *in vivo* biodistribution, target engagement, and pharmacokinetics, supporting both immunotherapy development and treatment monitoring.

Looking beyond diagnosis, Flögel and colleagues [8] explore the concept of targeted fluorine-19 MRI for molecular theranostics. Their work illustrates the evolving role of imaging, from visualizing immune responses to enabling targeted therapeutic interventions. Overall, these studies reflect an important shift in the field from imaging as a descriptive technology towards a quantitative tool that can inform therapeutic decision-making and support precision medicine.

## Looking ahead

Despite considerable progress, the full potential of molecular imaging in immunotherapy is only beginning to be realized. Emerging technologies such as total-body PET [9] offer new opportunities to study immune cell trafficking, therapeutic biodistribution, and interactions between multiple organs at the whole-body level, while developments in molecular and cellular imaging continue to provide increasingly detailed information at finer biological scales. Future advances will increasingly depend on integrating these complementary imaging approaches with spatial biology, multi-omics, computational modelling, and artificial intelligence to generate quantitative, multiscale representations of immune responses. Their integration offers an opportunity to connect molecular and cellular events with tissue-level changes and whole-body physiology, providing a more complete picture of how immune responses evolve during disease and treatment [2].

Equally important will be the development of quantitative imaging biomarkers supported by standardized acquisition protocols, harmonized analytical pipelines, and reproducible validation across institutions. Community-driven initiatives and international consortia such as Immunemap, CGT-TRACS, and Immune-Image, together with collaborative research infrastructures such as Euro-BioImaging and the National PET Imaging Platform (NPIP), will play an important role in improving access to advanced imaging technologies, promoting data sharing and cross-study comparability, and supporting multicentre translational studies.

Technological innovation alone, however, will not ensure clinical adoption. Successful implementation will require close collaboration between academia, healthcare providers, industry, imaging scientists, and regulatory agencies. Practical considerations including regulatory approval, cost-effectiveness, reimbursement, and integration into routine clinical workflows will need to be addressed if promising imaging technologies are to become accessible tools for patient care.

This Special Collection highlights the breadth and growing applications of molecular imaging across immunology and immunotherapy, from investigating fundamental immune processes to disease characterization, treatment monitoring, and image-guided therapeutic strategies. By bridging molecules, cells, tissues, and whole-body physiology, imaging has the potential to deepen our understanding of immune responses while transforming how immunotherapies are developed, evaluated, and delivered. As the field continues to evolve, seeing the ‘big picture’ of immunity across space and time will be central to understanding the mechanisms that determine response and resistance to immunotherapy.

## Data Availability

Not applicable.
